# Prognostic Significance of Infarct Size and Location: The Case of Insular Stroke

**DOI:** 10.1038/s41598-018-27883-3

**Published:** 2018-06-22

**Authors:** Carlos Laredo, Yashu Zhao, Salvatore Rudilosso, Arturo Renú, José Carlos Pariente, Ángel Chamorro, Xabier Urra

**Affiliations:** 1 Functional Unit of Cerebrovascular Diseases, Hospital Clinic, Barcelona, Spain; 2 Institut d’Investigacions Biomèdiques August Pi i Sunyer, Barcelona, Spain; 3 Medicine Department, School of Medicine, Universitat de Barcelona, Barcelona, Spain

## Abstract

The prognostic relevance of strokes in different locations is debated. For example, insular strokes have been associated with increased mortality, but this association could reflect their greater severity. In two independent cohorts of patients with supratentorial ischemic stroke (n = 90 and 105), we studied the prognostic consequences of lesion size and location using voxel-based lesion-symptom mapping before and after volume control, which better accounts for total lesion volume. Strokes affecting the insula were larger than non-insular strokes (28 vs 2cc and 25 vs 3cc, p < 0.001 in both cohorts). A number of supratentorial areas (mainly in the left hemisphere), including the insula, were associated with poor functional outcome in both cohorts before (4014 voxels) and after volume control (1378 voxels), while the associations with death were greatly reduced after volume control (from 8716 to 325 voxels). Exploratory analyses suggested that the method of lesion volume quantification, the National Institutes of Health Stroke Scale hemispheric bias and patient selection can result in false associations between specific brain lesions and outcomes. In conclusion, death in the first months after stroke is mainly explained by large infarct volumes, whereas lesions of specific supratentorial structures, mostly in the left hemisphere, also contribute to poor functional outcomes.

## Introduction

The brain is a complex organ with heterogeneous cytoarchitectural areas that have been distinguished for long^1^. Recent studies based on modern neuroimaging data have expanded the parcellation of the human brain cortex^2^. It is, therefore, natural to think that certain brain lesions may cause specific neurological deficits^3^ and also that some brain lesions may be associated with worse outcomes than others. The study of the insula has triggered special interest due to the implications of this region in multiple brain functions, many related to the autonomic nervous system, subjective feelings, emotions, and self-awareness by receiving information on the state of the body^4^. Lesions affecting the insula have been related to assorted consequences, including disruption of smoking addiction^5^, also after stroke^6^. Experimental stimulation of the insula can raise plasma catecholamine concentrations, cause myocardial damage, and increase the incidence of cardiac arrhythmias^7–9^. Accordingly, strokes involving the insula, have been associated with diverse complications (Supplementary eTable 1), including autonomic dysfunction with altered heart rate variability^10–12^, adverse cardiac events^13^, troponin elevations^14^ and incident arrhythmias^15–18^, immunodepression^19^, infections^19–21^, dysphagia^22^, hyperglycemia^23^, poor functional outcomes^24^, sudden death^10^ and total mortality^25–28^. In many^10–12,14,15,18,23,25–28^, but not all^13,17,21^, of these studies, the associations occurred with right insular lesions. However, other studies did not find a specific role of the insula in the emergence of these endpoints^29–33^, which led some investigators to argue that these associations resulted from the greater severity of insular strokes^34^. Indeed, the size of the stroke is a great predictor of poor outcome^35–37^, and strokes affecting the insula are large due to the vascular anatomy of the brain^28,38,39^.

Overall, there are conflicting results in the literature regarding the prognostic relevance of lesions in different brain areas, including the involvement and laterality of insular lesions. Recent studies have reopened the discussion on the clinical relevance of insular involvement in stroke patients^25,40–42^, suggesting that the use of variable techniques to adjust for the influence of infarct size could explain some of the current discrepancies. Here, we tried to overcome some of the limitations of previous reports on the contribution of stroke size and specific supratentorial lesions to death and poor functional outcome. For this, in two independent cohorts of patients we used conventional voxel-based lesion symptom mapping (VLSM) in magnetic resonance imaging (MRI) images, and also VLSM after volume control (VC), to better account for the effects related to total lesion size^43^. As a result, we were able to describe the contribution of the size of the stroke and the lesion of specific brain areas to the occurrence of death and poor functional outcome in patients with ischemic stroke.

## Material and Methods

### Subjects

The cohort 1 (C1) included all patients with supratentorial ischemic strokes in the “Immunological Biomarkers in Patients with Acute Ischemic Stroke” study (Clinicaltrials.gov identifier NCT01894529)^44^. The cohort 2 (C2) included patients from the local revascularization database at Hospital Clínic that had a supratentorial lesion and a full imaging workup. The patients were admitted to a dedicated stroke unit, and the neurological course was assessed using the National Institutes of Health Stroke Scale (NIHSS). Trained stroke neurologists assessed death and functional outcome at three months after stroke, and we defined poor functional outcome as a modified Rankin Scale (mRS) score >2. We categorized the cause of death as cerebrovascular (fatal swelling of the infarct, fatal intracranial hemorrhage, death from initial stroke or fatal recurrent stroke), cardiovascular, infections or unknown as previously described^25^. Other clinical and radiological variables included: age, sex, glucose levels at admission, the presence of intracranial large vessel occlusion (up to M2 segments of the middle cerebral artery), the type of acute revascularization treatment, and etiologic subtype. The research was conducted according to the principles of the Declaration of Helsinki, and all the patients or their legal representatives provided written informed consent and approved storage of their data in a local database for the purpose of research that was declared into a Web-based registry for monitoring by the Catalan Health Department that satisfied all legal requirements for protection of personal data. The Clinical Research Ethics Committee at Hospital Clínic approved the study (registration number HCB/2017/0811).

### MRI data Acquisition and Lesion Symptom Mapping Analysis

MR imaging was performed on a 1.5 T Siemens MAGNETOM Aera scanner (Siemens, Erlangen, Germany) with a median delay of 1 day after stroke onset in both cohorts. The stroke MRI protocol included a diffusion-weighted image (DWI) sequence obtained with a voxel size of 2 × 2 × 5 mm^3^ and a structural T1-weighted image with a voxel size of 1 × 1 × 5 mm^3^. Brain infarcts were segmented and quantified on anonymized scans selecting DWI regions with a signal intensity exceeding by >3 standard deviations the intensity of the contralateral hemisphere (Supplementary eFig. 1a). T1-weighted images were transferred into stereotaxic space using the DARTEL normalization algorithm in SPM8, and the transformation was then applied to DWI and acute lesion masks to place all the images in the Montreal Neurological Institute space for further analysis (Supplementary eFig. 1b). To increase the statistical power of the study, we did the primary analysis after flipping the images to the same hemisphere. We assessed the effect of laterality without flipping the images and repeated the bilateral analysis in a merged cohort including all 195 patients (Supplementary eFig. 1c). In descriptive tables, patients with bilateral infarcts (n = 9) were categorized according to the hemisphere with the greatest infarct volume. For each VSLM analysis (C1, C2 or merged cohorts), we included voxels damaged in at least 10% of the subjects. We used the Liebermeister test to study the association between the presence of lesions and the different clinical outcomes (death and poor functional outcome). For controlling the effect of total infarct volume, we studied the association between affected voxels and the outcomes in a general linear model in which we placed greater weight on lesioned voxels when the total lesion volume was smaller. For this, the lesion status of each subject was either 0 (no lesion on the voxel) or the reciprocal of the norm of the lesion map [(1/(square root of total lesion volume)]^43^. Family-wise error rates were avoided using repeated permutation tests (4000 permutations, p = 0.01) to control for multiple comparisons^45^. We show the voxels with supra-threshold z-scores (Supplementary eFig. 1d) and overlaid the voxels on the Automated Anatomical Labeling atlas^46^ or the Johns Hopkins University white-matter tractography atlas^47^ to determine the damaged cortical regions and white-matter tracts (Supplementary eFig. 1e).

### Statistical analysis

We reported continuous variables as mean with standard deviation or median with interquartile ranges and compared them with the Student t-test or Mann–Whitney test as appropriate. We compared categorical variables with the chi-square and Fisher exact tests. We assessed the independent effect of insular lesion over the risk of death and poor functional outcome in logistic regression models that included clinical variables associated with the outcomes in univariate comparisons. The independent effect of baseline clinical severity (NIHSS score) and infarct volume was assessed in different models to avoid the collinearity of these variables. We compared the predictive value of infarct volume, NIHSS score, and age over death and poor functional outcome using Receiver Operating Characteristic curves. Although the power of the study to detect associations between the lesion of specific structures and the clinical endpoints using conventional statistics is limited (Supplementary eTable 2), especially for endpoints with low prevalence or structures lesioned in few patients, the voxel-based analysis is more powerful and requires controlling for multiple comparisons to avoid false positive results. We performed all tests using SPSS (v22; IBM, Armonk, NY) and established the level of significance at the 0.05 level (2-sided).

## Results

### Insular strokes are clinically more severe at onset, larger, and associated with poor functional outcomes at follow up

**Figure 1:**
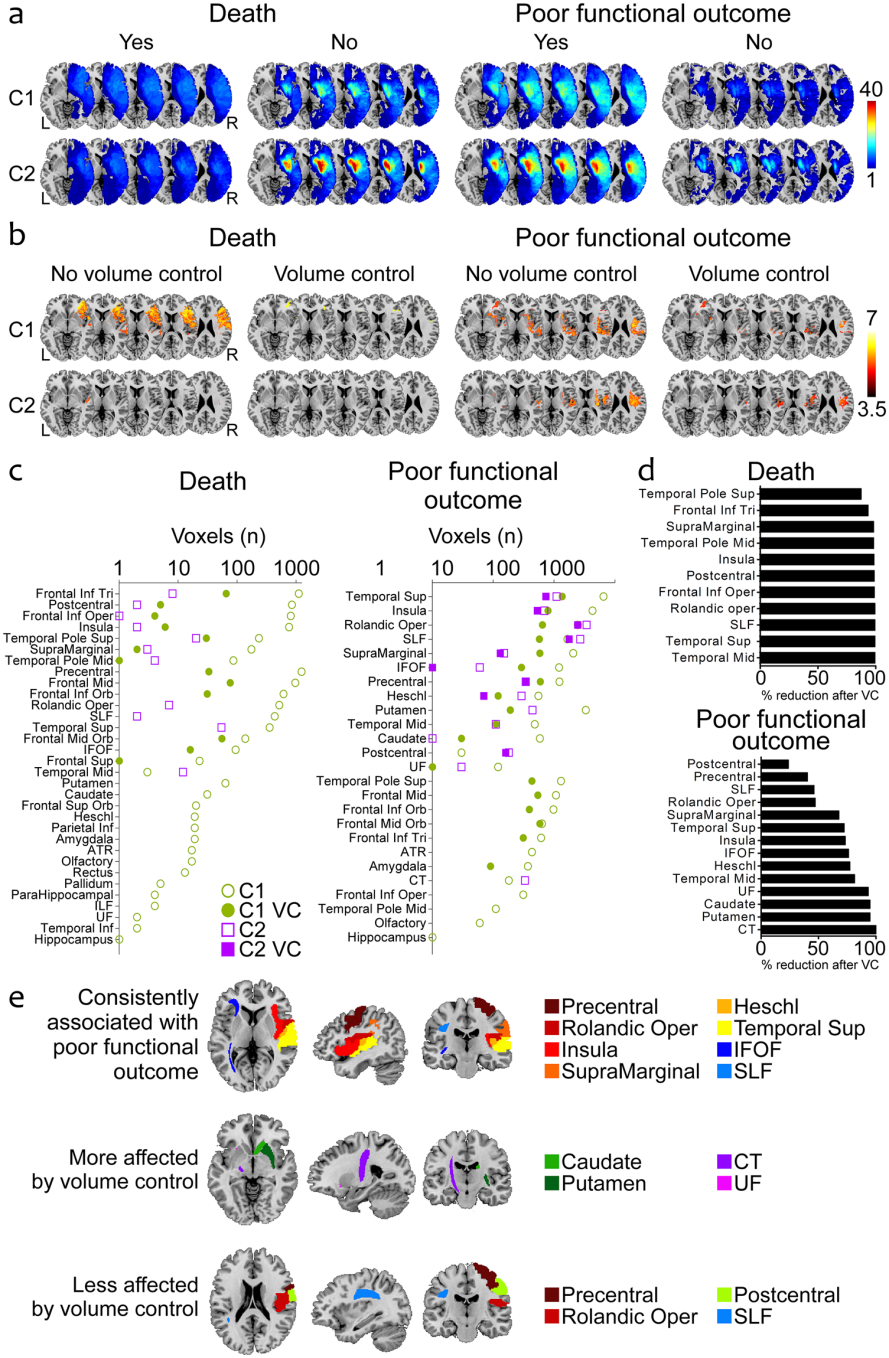
Unilateral analysis of the associations between lesions in different supratentorial regions and 3-month outcomes. (**a**) Lesion overlays of patients with or without poor functional outcome and death in C1 and C2. (**b**) Results of VLSM analysis: z-score maps revealing significant associations with mortality and poor functional outcome before and after VC in C1 and C2. (**c**) Number of voxels in each anatomical region associated with death and poor functional outcome before and after VC in C1 and C2. (**d**) Percentages of voxels associated with each outcome measure in C1 and C2 that were absent after doing the VC analyses. The graphics only include brain regions that were associated with death or poor functional outcome before VC in both C1 and C2. (**e**) Anatomic representation of cortical areas and white matter tracts: consistently associated with poor functional outcome in all 4 analyses (C1, C2, with or without VC); whose association with poor outcome was most reduced after VC; with the least reductions in the associations with poor outcome after VC. Abbreviations: VC, volume control; C1, cohort 1; C2, cohort 2; SLF, superior longitudinal fasciculus; IFOF, inferior fronto-occipital fasciculus; UF, uncinate fasciculus; ATR, anterior thalamic radiation; CT, corticospinal tract; ILF, inferior longitudinal fasciculus.

In both cohorts, patients with insular strokes (63% and 70% in C1 and C2, respectively) had more severe strokes both at admission and at follow-up, larger lesions, poorer functional outcomes and greater death rates at three months (Table 1). The main cause of death in the first three months after stroke was cerebrovascular. The lesion overlays in both C1 and C2 showed that patients with poor outcomes had larger lesions compared with patients with better outcomes, and this was particularly apparent in the case of death: the lesions were homogeneously distributed over the entire hemispheric territory in patients who died (Fig. 1a).

**Table 1:** General characteristic of patients in both cohorts.

	COHORT 1			P	COHORT 2			P
	All patients (n = 90)	Insula+ (n = 57)	Insula− (n = 33)		All patients (n = 105)	Insula+ (n = 74)	Insula− (n = 31)	
Age (years), mean (SD)	73 (12)	74 (13)	71 (12)	0.248	69 (12)	68 (12)	71 (11)	0.252
Sex (male), %	63	60	70	0.340	51	51	52	0.980
Admission NIHSS score, median (IQR)	10 (4–17)	14 (9–20)	4 (2–8)	<0.001	17 (12–20)	17 (12–20)	12 (10–18)	0.017
Glucose at admission,	138 (56)	131 (41)	149 (75)	0.162	128 (39)	129 (43)	126 (29)	0.754
Large vessel occlusion, %	83	89	71	0.005	96	97	94	0.580
Acute revascularization, %				0.339				0.273
None	15	12	18		5	3	10	
IV only	62	60	67		24	26	19	
IA (±IV)	23	28	15		71	71	71	
24 h NIHSS score, median (IQR)	5 (2–15)	9 (4–19)	2 (0–3)	<0.001	8 (3–16)	10 (5–17)	4 (1–12)	0.001
Infarct volume (cc), median (IQR)	18 (3–53)	28 (17–82)	2 (1–6)	<0.001	15 (5–53)	25 (11–82)	3 (0.2–10)	<0.001
Laterality (right/left), %	48/52	53/47	41/59	0.277	48/52	50/50	42/58	0.450
Etiologic stroke subtype, %				0.017				0.022
Cardioembolic	43	51	29		50	53	36	
Large vessel atherosclerosis	19	14	29		18	16	24	
Lacunar	6	0	17		0	0	7	
Undetermined etiology	27	28	25		23	25	24	
Other	5	7	0		9	6	9	
3-month mRS score, median (IQR)	2 (1–4)	2 (1–4)	1 (0–2)	<0.001	3 (1–4)	3 (2–5)	1 (0–2)	0.135
Death at 3 months, %	12	18	3	0.050	11	15	3	0.105
Cause of death				0.231				0.007
Cerebrovascular	55	60	0		50	55	0	
Cardiovascular	18	20	0		17	18	0	
Infection	0	0	0		25	27	0	
Unknown	27	20	100		8	0	100	

Abbreviations: NIHSS, National Institutes of Health Stroke Scale; mRS, modified Rankin Scale.

### Controlling for volume altered the associations between different brain regions and outcomes, especially in the case of death

The VLSM analysis revealed that the extension of brain regions and the number of voxels associated with death (8716 voxels) and poor functional outcome (4014 voxels) decreased to 325 and 1378 voxels respectively after VC (Fig. 1b,c). Besides the insula, the superior temporal gyrus, the rolandic operculum, the superior longitudinal fasciculus, the supra-marginal gyrus and the inferior fronto-occipital fasciculus were associated with poor functional outcome in both cohorts before and after VC (Fig. 1c,e). The VC analysis reduced almost entirely the association with poor functional outcome of structures such as the corticospinal tract, the putamen, the caudate nucleus and the uncinate fasciculus, and affected the least the postcentral gyrus, the precentral gyrus, the superior longitudinal fasciculus and the rolandic operculum (Fig. 1d,e, Supplementary eTable 3). The insula was moderately affected by VC. The associations with death were less consistent and substantially reduced in all the brain regions after introducing VC (Fig. 1c,d).

### The effect of laterality over poor functional outcome after stroke

**Figure 2:**
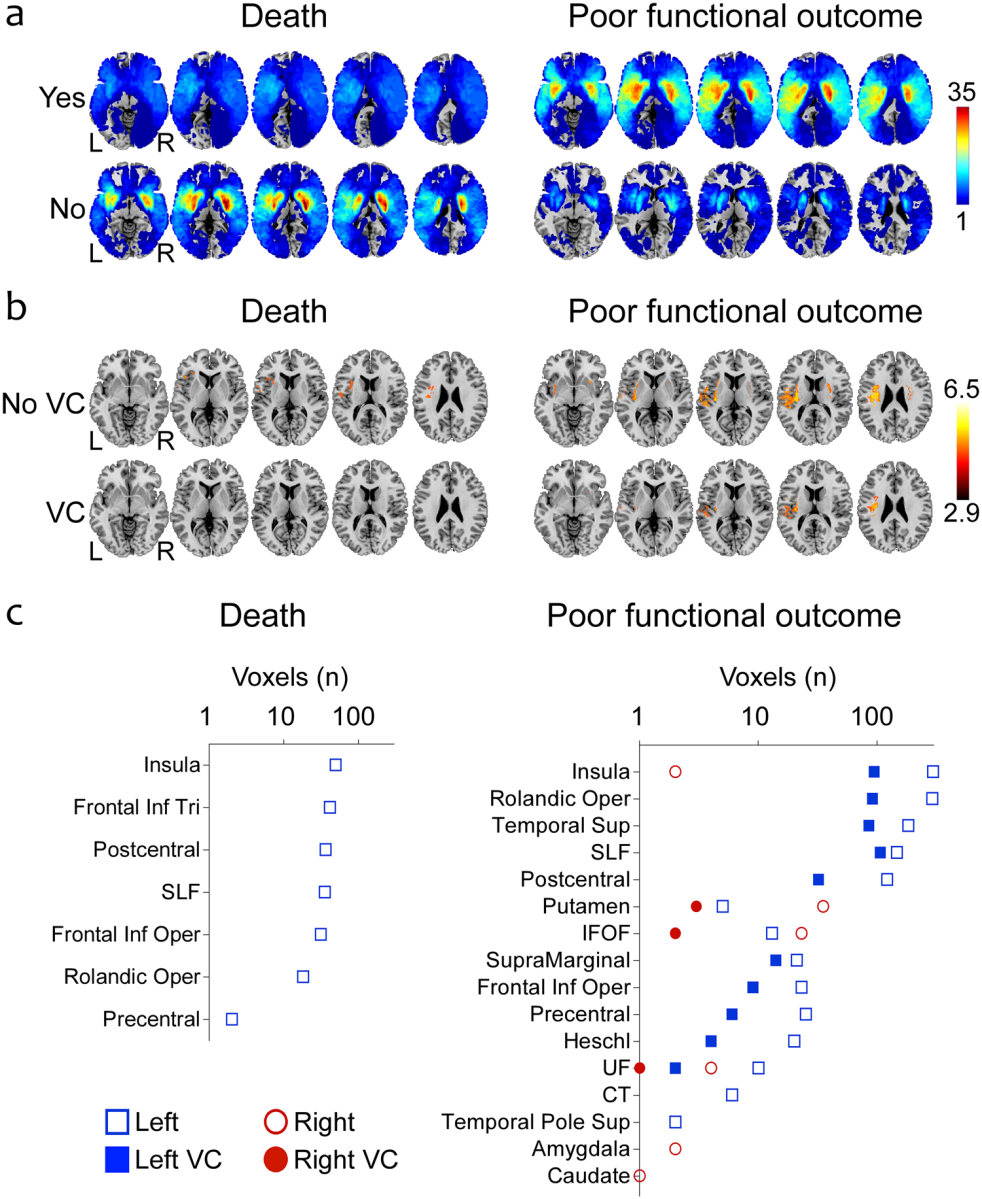
Effect of lesion laterality: bilateral analysis of the associations between lesions in different supratentorial regions and 3-month outcomes. (**a**) Lesion overlays of patients with or without poor functional outcome and death in the merged C1 + C2 cohort. (**b**) Results of VLSM analysis: z-score maps revealing significant associations with death and poor functional outcome before and after VC in the merged C1 + C2 cohort. (**c**) Number of voxels in each anatomical region associated with death and poor functional outcome before and after VC in the merged C1 + C2 cohort: the associations mostly affect left brain regions. Abbreviations: VC, volume control; SLF, superior longitudinal fasciculus; IFOF, inferior fronto-occipital fasciculus; UF, uncinate fasciculus; CT, corticospinal tract.

In both cohorts, poor functional outcome was associated with few areas in the left hemisphere (Supplementary eFig. 2), and we found no associations with death. In the merged cohort (n = 195), patients with right and left hemispheric lesions had similar characteristics (Fig. 2a) except for greater NIHSS scores and higher glucose levels in patients with left lesions (Table 2). In VLSM analysis, the associations with death were very few (212 voxels) and disappeared after VC (Fig. 2b,c), while poor functional outcome was mostly associated with left lesions: 1158 (before VC) and 443 voxels (after VC) in the left hemisphere vs only 67 (before VC) and 6 voxels (after VC) in the right hemisphere. The voxels associated with poor outcome were mainly located in the insula, the rolandic operculum, the superior temporal gyrus and the superior longitudinal fasciculus (Fig. 2b,c).

**Table 2:** General characteristics of the whole population of 195 patients according to the laterality of the lesion.

	Right (n = 94)	Left (n = 101)	P
Age (years), mean (SD)	71 (12)	71 (12)	0.904
Sex (male), %	57	56	0.887
Admission NIHSS score, median (IQR)	12 (10–17)	16 (8–21)	0.003
Glucose at admission,	125 (36)	140 (56)	0.033
Large vessel occlusion, %	81	85	0.249
24 h NIHSS score, median (IQR)	6 (2–12)	7 (2–19)	0.117
Infarct volume (cc), median (IQR)	18 (6–45)	17 (3–54)	0.863
Insular stroke, %	72	64	0.197
3-month mRS score, median (IQR)	2 (1–4)	2 (1–4)	0.681
Death at 3 months, %	10	14	0.354

Abbreviations: NIHSS, National Institutes of Health Stroke Scale; mRS, modified Rankin Scale.

### Bias in patient selection and inaccurate lesion size measurements can influence the predicted associations between brain lesions and outcomes

**Figure 3:**
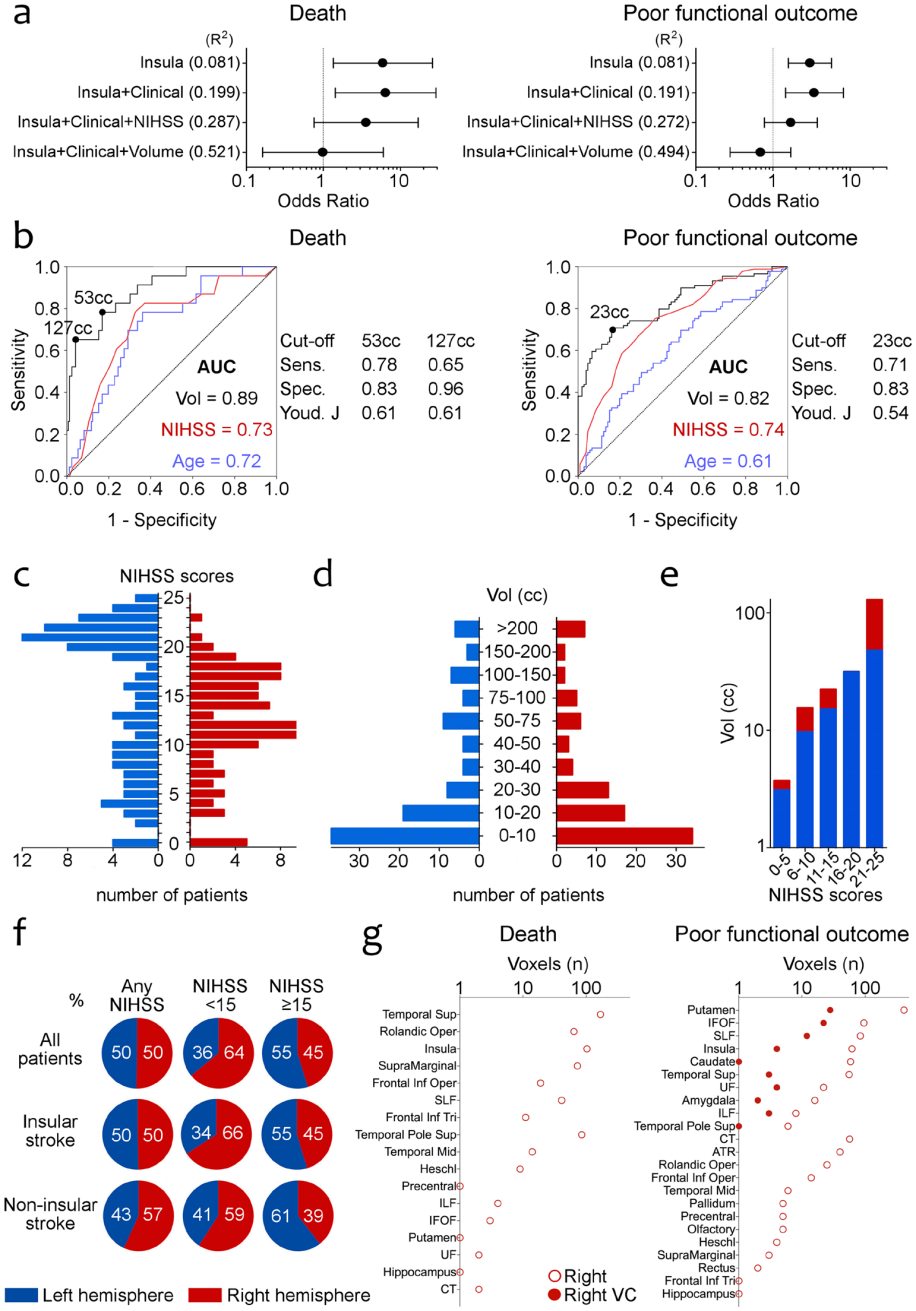
Inaccurate lesion volume measurements and patient selection can influence the laterality of associations with outcome measures. (**a**) Forest plots showing the odds ratios of death and poor functional outcome in insular strokes in different logistic regression models. (**b**) Receiver Operating Characteristic curves of infarct volume, NIHSS score and age for death and poor functional outcome and the performance of different volume cut-off points. Infarct volume had the greatest predictive value, specially for mortality. (**c**) The distribution of the number of patients with left and right hemispheric lesions in each NIHSS score at admission is asymmetrical. (**d**) The number of patients in each infarct size category is similar in right and left hemispheric lesions. (**e**) Infarct volumes are greater in right hemispheric lesions compared to left lesions in each NIHSS score category. (**f**) Total infarct volume in the whole cohort is similar in right and left lesions, but right lesions are greater in patients with lower NIHSS scores (especially in insular strokes) while left hemispheric lesions prevail in higher NIHSS scores. (**g**) Right brain structures are associated with death and poor functional outcome in VLSM analysis of a biased population after deleting patients with left hemispheric lesions in the upper NIHSS tertile. Abbreviations: NIHSS, National Institutes of Health Stroke Scale; AUC: area under the curve; Vol, volume; Sens., sensitivity; Spec., specificity; Youd. J, Youden’s J statistic; SLF, superior longitudinal fasciculus; IFOF, inferior fronto-occipital fasciculus; UF, uncinate fasciculus; ATR, anterior thalamic radiation; CT, corticospinal tract; ILF, inferior longitudinal fasciculus; VC, volume control.

We finally made exploratory analyses to explain the discrepancies in the literature regarding the clinical relevance of right and left insular lesions. Standard statistical analyses suggest that infarct volume greatly influences the risk of death and poor functional outcome (Fig. 3a,b). For example, the OR of death with insular involvement decreased from 5.92 (95%CI 1.34–26.09) in unadjusted models to 0.99 (95%CI 0.16–5.97) in models adjusted for clinical variables (NIHSS score and age) and infarct volume. Similarly, the OR of poor outcome with insular involvement decreased from 3.02 (95%CI 1.59–5.76) to 0.69 (95%CI 0.28–1.71). Patients with left hemispheric strokes were skewed towards higher NIHSS scores (Fig. 3c) while the distribution of patients with right and left hemispheric strokes along different infarct volume categories was almost identical (Fig. 3d). As a result, at the same NIHSS scores right hemispheric lesions tended to be larger than left hemispheric lesions (Fig. 3e). Stratifying the populations within specific NIHSS scores resulted in a predominance of larger right lesions in groups of patients with lower NIHSS scores, and a predominance of left lesions in patients with higher NIHSS scores. To assess the effect of patient selection bias, we constructed a biased population by deleting the 33 patients with left hemispheric lesions in the upper NIHSS tertile (scores 21 to 25). In this population baseline demographic and clinical characteristics, including the NIHSS score, were balanced in patients with right and left hemispheric lesions, but lesion size was significantly greater in patients with right lesions (Supplementary eTable 4). The VLSM analysis showed associations with right brain areas that were almost completely (although not entirely) reduced after VC (Fig. 3g).

## Discussion

By combining accurate measurements of ischemic lesion volumes and an imaging analysis methodology that allows studying the associations between lesion topography and different outcome measures, we were able to demonstrate the huge prognostic impact of lesion size in stroke. This was particularly apparent in the case of mortality, which was almost exclusively explained by the size of the lesion. Multiple reasons can explain why larger infarcts cause poor functional outcomes. Besides the fact that large lesions affect multiple different brain areas with specific functions, some consequences of stroke may result from the involvement of anatomical structures integrated into complex functional networks, and this may happen more often in large strokes^3,48,49^. It is possible that the brain regions implicated in the neurological deficits after acute brain injury are greater than what the results of lesion symptom mapping studies suggest, because damage to areas involved in relevant functional networks may cause further neurological deficits^50^. This is clearly suggested by the association between the superior longitudinal fasciculus (particularly the left) and poor functional outcome, since this tract is involved in brain functions such as attention, memory, emotions and language^51^. We assessed mortality at three months and, as in other recent reports^25^, the main cause of death in both cohorts was cerebrovascular. We cannot rule out the possibility that at the long-term, lesions of the insula could be associated with recurrent stroke or cardiac events, but these results stress that in the first months after stroke, mortality is mainly due to complications of large infarcts. The VC approach reduced the extent of the regions associated with poor functional outcome confirming the great importance of infarct volume^52^. This reduction was especially apparent in deep structures, but also in cortical regions of the temporal lobe and the insula, that are overrepresented in patients with large ischemic lesions in the territory of the middle cerebral artery^34,39,53^. It is possible that the VC analysis penalized too much the relevance of some of these areas in the brain. However, we preferred to use a restrictive approach stressing the relevance of brain areas that were associated with the outcomes in all types of analysis in both cohorts of patients. The similarities in the results in C1 and C2 despite baseline differences in the general characteristics of the patients supports the reliability of the imaging analysis.

The use of the mRS to define poor functional outcome could explain the link between functional impairment and certain brain areas in the left hemisphere, since deficits caused by left lesions, such as aphasia, are associated with even poorer outcomes compared to hemiparesis when using the mRS^54^. Besides the left superior longitudinal fasciculus, other left structures associated with poor functional outcomes even after considering the effect of total lesion volume were the insula, the rolandic operculum, and the superior temporal gyrus. The insula may be associated with poor outcomes through various mechanisms, including dysautonomia, cardiac complications or greater risk of stroke-associated infections, although the association of these complications with the left insula was not consistent in the past^13,44,55^. Lesions of the left superior temporal gyrus may cause disability due to its implication in speech comprehension and production^56,57^. Lesions in the left rolandic operculum have also been linked to subtle alterations in the production of speech after ischemic lesions^58^ and to prolonged dysphagia, although a clear lateralization was not found in the latter^59^.

The link between left hemispheric lesions and poor functional outcomes is also consistent with the results of several studies that used lesion symptom mapping^52,60,61^, but differs from a large number of studies that described associations of different outcomes with right insular lesions. In the Supplementary eTable 1 we reviewed the methodological heterogeneity of the studies investigating the role of the insula: many of them did not measure infarct size or categorized it^11,12,16,25^, or used the NIHSS for matching patients^20^ or creating subgroups of patients^40^, and this approach may not be optimal for lesion mapping. Our results suggest that inaccurate measure or control of total lesion volumes biased populations of patients and can result in overestimations of the role of right insular lesions. Regarding the NIHSS, in a principal components factor analysis, the two main factors underlying the NIHSS score are the right and left hemispheres^62^. As in our study population, left hemispheric lesions typically score 4 points more than right lesions^62–64^. For that reason, severity scoring must include the side of the infarct^62^ to avoid overestimating the role of right-sided structures. Furthermore, dividing populations by NIHSS scores can result in larger right infarcts in the group of patients with lower NIHSS, and this was more apparent in the subgroup of patients with insular infarcts (Fig. 3f). Quantification of the infarct volume seems essential and preferable to using the NIHSS as a surrogate measure of infarct size in every study aimed at localizing specific symptoms or consequences of stroke. Reassuringly, even in the biased population, the VC analysis was able to almost completely avoid the false associations with right regions (Fig. 3g). Even when infarct volumes are quantified, the statistical analysis can greatly influence the results. For example, the inclusion of two highly collinear variables such as the NIHSS and infarct volume in the same multivariable tests^23^ could favor associations with right structures. Methods of automatic variable selection in regression analysis can result in errors if they skip crucial variables^65^, such as infarct volume, in populations where right and left strokes have different sizes^26^. Why should hemispheric laterality influence the size of the stroke? In our entire population patients with right and left hemispheric strokes had exactly the same infarct volumes (Fig. 3f), but in several reports right insular strokes were larger than left insular strokes^26,28^, probably due to lower recognition of mild right hemispheric strokes^66^ or to difficulties in obtaining consent for research from patients with large left hemispheric strokes^26^.

The main limitations of this study are its retrospective nature and the use of a broad and a relatively weak (as compared with death) definition of poor functional outcome. The mRS is widely used and that facilitates the generalizability of these results, but the definition of poor outcome based on this scale may have favored associations with some left hemispheric structures, while other brain areas may be linked to other specific deficits and complications that are less reflected in the mRS. Finally, despite the solid information that lesion mapping studies give on the prognostic relevance of infarct size and location, studying strategic infarcts may still be necessary to understand the neurological consequences of specific brain lesions. For example, strokes restricted to the insular cortex have been related to many different symptoms, including hypertensive episodes in right lesions^67^, suggesting that right insular lesions may indeed cause autonomic disturbances. The main drawback of this approach is the difficulty in recruiting enough patients with strategic infarcts, but studies involving very large numbers of patients may allow even lesion mapping analysis in patients with small infarcts located in a distributed manner.

In conclusion, this study highlighted the fundamental importance of the volume of ischemic injury in determining death and poor outcome, which was almost exclusively linked to infarct size. Due to this strong prognostic effect of lesion size, it is necessary to adequately measure it in all studies investigating the prognostic consequences of brain lesions. It also suggested an independent association of certain brain regions, especially in the left hemisphere, with poor functional outcomes after ischemic stroke. The association was evident even after a rigorous control of the effect of total lesion volume.

## Electronic supplementary material

Supplementary Figures and Tables
