# Supplementary material for: Prognostic Significance of Infarct Size and Location: The Case of Insular Stroke
Source: Sci Rep. 2018 Jun 22;8:9498. doi: 10.1038/s41598-018-27883-3 (PMC6015086; doi:10.1038/s41598-018-27883-3)
Supplement: Supplementary file 1 — Supplementary Figures and Tables [file 41598_2018_27883_MOESM1_ESM.pdf]

Supplementary information

PROGNOSTIC SIGNIFICANCE OF INFARCT SIZE AND LOCATION: THE CASE OF INSULAR STROKE

Carlos Laredo MSc[1*]; Yashu Zhao MD[1*]; Salvatore Rudilosso MD[1]; Arturo Renú MD, PhD[1]; José Carlos Pariente MSc[2]; Ángel Chamorro MD, PhD[1,2,3] Xabier Urra MD, PhD[1,2]

Affiliations:

[1] Functional Unit of Cerebrovascular Diseases, Hospital Clinic, Barcelona, Spain

[2] Institut d'Investigacions Biomèdiques August Pi i Sunyer, Barcelona, Spain

[3] Medicine Department, School of Medicine, Universitat de Barcelona, Barcelona, Spain

*C.L. and Y.Z. contributed equally to this manuscript

Corresponding authors:

Ángel Chamorro, Functional Unit of Cerebrovascular Diseases, Hospital Clínic, 170 Villarroel, 08036 Barcelona, Spain; Phone: +34 93 227 54 14; Fax: +34 93 227 57 89; Email: achamorro@clinic.cat.

Xabier Urra, Functional Unit of Cerebrovascular Diseases, Hospital Clínic, 170 Villarroel, 08036 Barcelona, Spain; Phone: +34 93 227 54 14; Fax: +34 93 227 57 89; Email: xurra@clinic.cat.

eFigure 1. Neuroimaging methods.

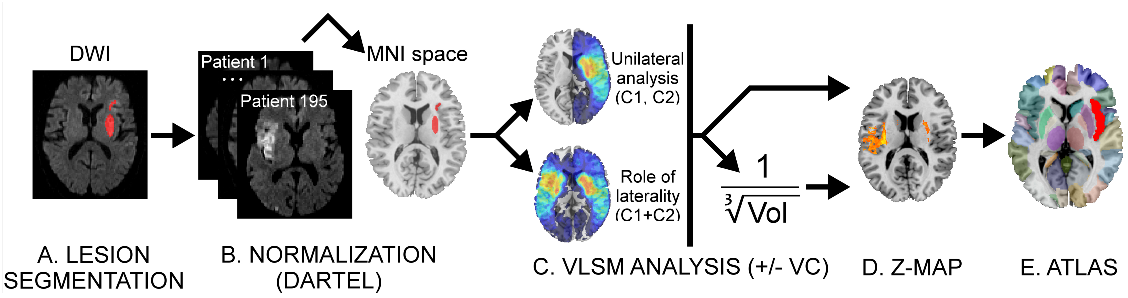

Abbreviations: DWI, diffusion-weighted image; MNI, Montreal Neurological Institute; VLSM, voxel-based lesion symptom mapping; Vol, volume.

eFigure 2. Bilateral analysis in C1 and C2: number of voxels in each anatomical region associated with poor functional outcome before and after VC.

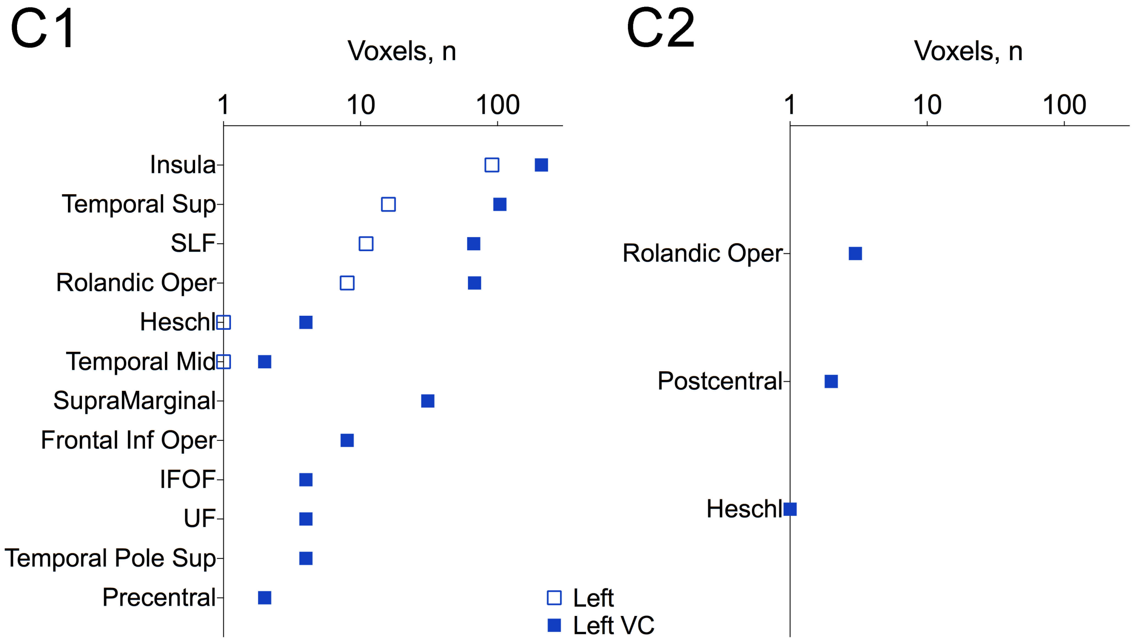

In both cohorts, poor functional outcome was only associated with lesions of left brain structures. There were no associations with death in the bilateral analysis in any of the cohorts. Abbreviations: C1, cohort 1; C2, cohort 2; SLF, superior longitudinal fasciculus; IFOF, inferior fronto-occipital fasciculus; UF, uncinate fasciculus; VC, volume control.

eTable 1. Imaging methods in studies that investigated the clinical relevance of insular infarcts and/or laterality of the stroke.

Main study outcome*	Authors	n	Association with insula	Imaging modality	Assessment of infarct size
Mortality	Hanne L, et al.[26]	736	Right insula	MRI	Quantitative (converted into tertiles), not used in the final statistical model
Mortality	Alves JN, et al.[41]	561	Insula, none after controlling for clinical severity	CT, MRI	None besides baseline ASPECTS score
Mortality	Sposato LA, et al.[25]	2099	Right insula	CT, MRI	Categorical (no visible, small/medium, large/very large)
Mortality	Borsody M, et al.[34]	111	Insula, none in adjusted models	CT, MRI	Semi-quantitative (A+B+C/2)
Mortality	Fink JN, et al.[63]	1644	No effect of laterality, but infarct location not studied	Not specified	None
Mortality (cardiac death)	Rincon F, et al.[33]	655	None, association with left parietal lobe	CT, MRI	Categorical (<1/2 lobe, ½-1 lobe, >1 lobe)

Mortality	Chamorro A, et al.[32]	136	None	CT, MRI	Semi-quantitative
Mortality	Abboud H, et al.[28]	510	Right insula	MRI	Semi-quantitative (A+B+C/2), not included in the model despite significant differentes in the volume of non-insular, left-insular and right-insular infarcts
Mortality	Christensen H, et al.[27]	179	Right insula	CT	Quantitative
Mortality	Colivicchi F, et al.[15]	208	Right insula	CT, MRI	Semi-quantitative (A+B+C/2), not used in the model
Mortality (sudden death)	Algra A, et al.[55]	2885	None, associated with left-sided infarcts	CT, MRI	Categorical (≥2 cm)
Mortality	Tokgözoglu SL, et al.[10]	62	Right insula	CT, MRI	Not specified
Cardiac (arrhythmias)	Seifert F, et al.[18]	93	Right hemispheric infarcts, including the insula, none in volume-adjusted models	CT, MRI	Quantitative, cleared the associations when included in the imaging analysis

Cardiac (adverse cardiac outcome)	Laowattana S, et al.[13]	116	Left insula	CT, MRI	None
Cardiac (acute cardiovascular events)	Fink JN, et al.[38]	150	None	MRI	Categorical
Cardiac (new atrial fibrillation)	Scheitz JF, et al.[17]	1823	Insula, no effect of laterality	Not specified	None
Cardiac (Troponin T elevation)	Ay H, et al.[14]	738	Right insula	MRI	Quantitative, but not used in the imaging analysis
Cardiac (recent and new atrial fibrillation)	Vingerhoets F, et al.[16]	1661	Parietoinsular cortex	CT	None
Poor functional outcome	Munsch F, et al.[61]	289	None in adjusted models	MRI	Quantitative, included in the statistical analysis

Poor functional outcome	Timpone VM, et al.[24]	55	Percentage of insular infarct in the subgroup of patients with small strokes	CT, MRI	Quantitative, but dichotomized in the statistical models
Poor functional outcome	Wu O, et al.[52]	490	Left hemispheric lesions (minor contribution of the left insula)	MRI	Quantitative, included in the imaging analysis
Poor functional outcome	Yassi N, et al.[31]	152	None, associations in other regions in the brain	MRI	Quantitative
Poor functional outcome	Cheng B, et al.[60]	101	Insula, no effect of laterality	MRI	Quantitative, not included in the imaging analysis
Stroke-associated infection	Urra X, et al.[44]	106	Insula, no significant effect of laterality	MRI	Quantitative, included in the imaging analysis
Stroke-associated infection (pneumonia)	Kemmling A, et al.[20]	430	Right insula	MRI	Quantitative, not included in the imaging analysis

Stroke-associated infection	Walter U, et al.[19]	384	Insula, no effect of laterality	CT, MRI	Categorical (<1.5cm, 1.5cm-33% MCA, 33-66% MCA,>66% MCA), not included in the final statistical analysis
Stroke-associated infection	Wartenbert K, et al.[68]	94	Insula, none in adjusted models.	MRI	Quantitative
Stroke-associated infection	Harms H, et al.[69]	63	No association with insula nor laterality	CT	Quantitative
Stroke-associated infection	Minnerup J, et al.[29]	591	No specific location, no effect of laterality	CT, MRI	Categorical (<1.5cm, intermediate, >5cm or 1/3 of MCA)
Stroke-associated infection (pneumonia)	Steinhagen V, et al.[21]	60	Insula, no effect of laterality	CT, MRI	Categorical (<33% MCA, 33-66% MCA,>66% MCA)
Aspiration	Galovic M, et al.[22]	94	Insula and frontal operculum, no differences in laterality	MRI	Quantitative

Autonomic (sympathetic activation)	Meyer S, et al.[12]	29	Right insula	MRI	None
Autonomic (heart rate variability)	Barron SA, et al.[11]	40	Right hemisphere linked to reduced parasympathetic innervation	CT	None
Hyperglycemia	Winder K, et al.[23]	229	Right insula	MRI	Quantitative, introduced together with NIHSS in the imaging analysis
Smoking cessation	Suñer-Soler R, et al.[6]	110	Insula, no effect of laterality	CT, MRI	Semi-quantitative (A+B+C/2)

* In studies assessing several outcomes we show the clinically most important one. Abbreviations: MCA, middle cerebral artery; ASPECTS, Alberta Stroke Program Early CT Score.

eTable 2. Statistically significant prevalences of the clinical end-points in patients with insular strokes in each cohort with an alpha of 0.05 and a power of 0.8.

			Death				Poor outcome			
Cohort	n	Prevalence of insular stroke	Prevalence in non-insular stroke	Significant lower prevalence in insular stroke	Significant upper prevalence in insular stroke	Actual prevalence in insular stroke	Prevalence in non-insular stroke	Significant lower prevalence in insular stroke	Significant upper prevalence in insular stroke	Actual prevalence in insular stroke
C1	90	0.633	0.03	Not powered	0.24	0.175	0.152	Not powered	0.44	0.526*
C2	105	0.705	0.032	Not powered	0.23	0.149	0.419	0.12	0.74	0.554
C1+ C2	195	0.672	0.031	Not powered	0.15	0.16*	0.281	0.093	0.50	0.542*

* Prevalences above the statistically significant limits compared to patients with non-insular stroke.

eTable 3. Effect of total lesion volume: percentage of voxels associated to each region in the brain that disappear after VC.

Structure	Poor Outcome (%)	Death (%)
Temporal Mid	81	100
Insula	73	99
Temporal Sup	72	100
SupraMarginal	68	99
Rolandic Oper	47	100
SLF	46	100
Postcentral	24	99
CT	100	
Frontal Inf Oper		100
Temporal Pole Mid		99
Caudate	95	
Putamen	95	
Frontal Inf Tri		94
UF	93	
Temporal Pole Sup		88
Heschl	77	
IFOF	76	
Precentral	40	

We quantified the reduction in the association in structures that showed associations before VC in both cohorts. Abbreviations: SLF, superior longitudinal fasciculus; CT, corticospinal tract; UF, uncinate fasciculus; IFOF, inferior fronto-occipital fasciculus; VC, volume control.

eTable 4. General characteristics of the biased population after excluding patients with left lesions in the upper tertile of NIHSS scores.

	Right (n=94)	Left (n=68)	P
Age (years), mean (SD)	71 (12)	72 (12)	0.649
Sex (male), %	57	62	0.543

Admission NIHSS score, median (IQR)	12 (10-17)	10 (6-17)	0.174
Glucose at admission,	125 (36)	141 (64)	0.044
Large vessel occlusion, %	81	76	0.237
24h NIHSS score, median (IQR)	6 (2-12)	4 (2-12)	0.225
Infarct volume (cc), median (IQR)	18 (6-45)	11 (1-31)	0.023
Insular stroke, %	72	53	0.013
3-month mRS score, median (IQR)	2 (1-4)	1 (0-3)	0.024
Death at 3 months, %	10	7	0.605

Abbreviations: NIHSS, National Institutes of Health Stroke Scale; mRS, modified Rankin Scale.

Supplementary References

68. Wartenberg KE, Stoll A, Funk A, Meyer A, Schmidt JM, Berrouschot J. Infection after acute ischemic stroke: risk factors, biomarkers, and outcome. *Stroke Res Treat*. 2011;2011(1):830614. doi:10.4061/2011/830614.

69. Harms H, Reimnitz P, Bohner G, et al. Influence of stroke localization on autonomic activation, immunodepression, and post-stroke infection. *Cerebrovasc Dis*. 2011;32(6):552-560. doi:10.1159/000331922.
